# Identification and validation of ferroptosis-related lncRNA signatures as a novel prognostic model for glioma

**DOI:** 10.3389/fgene.2022.927142

**Published:** 2022-09-20

**Authors:** Liang Huang, Juan Zhang, Fanghua Gong, Yuhua Han, Xing Huang, Wanxiang Luo, Huaan Cai, Fan Zhang

**Affiliations:** ^1^ Department of Rehabilitation Medicine, Hunan Provincial People’s Hospital, The First Affiliated Hospital of Hunan Normal University, Changsha, Hunan, China; ^2^ Department of Nursing, Hunan Provincial People’s Hospital, The First Affiliated Hospital of Hunan Normal University, Changsha, Hunan, China; ^3^ Department of Cadre Health Care, Hunan Provincial People’s Hospital, The First Affiliated Hospital of Hunan Normal University, Changsha, Hunan, China; ^4^ Department of General Surgery, Hunan Provincial People’s Hospital, The First Affiliated Hospital of Hunan Normal University, Changsha, Hunan, China

**Keywords:** lncRNA, ferroptosis, glioma, prognostic signature, immune microenvironment

## Abstract

**Background:** Ferroptosis is a newly discovered form of regulated cell death with distinct properties and recognizing functions involved in physical conditions or various diseases, including cancers. However, the relationship between gliomas and ferroptosis-related lncRNAs (FRLs) remains unclear.

**Methods:** We collected a total of 1850 samples from The Cancer Genome Atlas (TCGA) and Genotype Tissue Expression (GTEX) databases, including 698 tumor and 1,152 normal samples. A list of ferroptosis-related genes was downloaded from the Ferrdb website. Differentially expressed FRLs (DEFRLS) were analyzed using the “limma” package in R software. Subsequently, prognosis-related FRLs were obtained by univariate Cox analysis. Finally, a prognostic model based on the 3 FRLs was constructed using Cox regression analysis with the least absolute shrinkage and selection operator (LASSO) algorithm. The prognostic power of the model was assessed using receiver operating characteristic (ROC) curve analysis and Kaplan-Meier (K-M) survival curve analysis. In addition, we further explored the relationship of the immune landscape and somatic mutations to prognostic model characteristics. Finally, we validated the function of LINC01426 *in vitro*.

**Results:** We successfully constructed a 3-FRLs signature and classified glioma patients into high-risk and low-risk groups based on the risk score calculated from this signature. Compared with traditional clinicopathological features [age, sex, grade, isocitrate dehydrogenase (IDH) status], the prognostic accuracy of this model is more stable and stronger. Additionally, the model had stable predictive power for overall survival over a 5-year period. In addition, we found significant differences between the two groups in cellular immunity, the numbers of many immune cells, including NK cells, CD4^+^, CD8^+^ T-cells, and macrophages, and the expression of many immune-related genes. Finally, the two groups were also significantly different at the level of somatic mutations, especially in glioma prognosis-related genes such as IDH1 and ATRX, with lower mutation rates in the high-risk group leading to poorer prognosis. Finally, we found that the ferroptosis process of glioma cells was inhibited after knocking down the expression of LINC01426.

**Conclusion:** The proposed 3-FRL signature is a promising biomarker for predicting prognostic features in glioma patients.

## 1 Introduction

Gliomas are the most common primary malignant brain tumors in adults, mainly in the brain and in glial tissue (Ostrom et al., 2013), accounting for 81% of malignant brain tumors. Although relatively rare relative to other cancers, they cause significant mortality and morbidity (Ostrom et al., 2014). Glioblastoma (GBM) is the most common and most clinically aggressive World Health Organization (WHO) grade IV glioma, with the highest degree of malignancy, the worst prognosis, and the lowest overall survival (OS) rate. The median OS of GBM is approximately 8 months, and the 5-year survival rate is 7.2% (Ostrom et al., 2020). Even with aggressive multimodal therapy, the median survival is only 12–15 months (Stupp et al., 2005). Glioma is also a highly heterogeneous tumor (Nicholson and Fine 2021; van den Bent et al., 2009), which makes it difficult to determine its prognostic effect and treatment response when treating glioma. Therefore, finding a biomarker and possible therapeutic target that can predict prognosis is crucial.

Ferroptosis is a novel cell death method first proposed in 2012 that is distinct from autophagy and apoptosis. Ferroptosis can be triggered by depleting the amino acid cysteine in the cell or by inhibiting the phospholipid hydroperoxidase glutathione peroxidase 4 (GPX4) (Dixon 2017). Ferroptosis is characterized by membrane lipid peroxidation in cells, which eventually leads to the loss of selective permeability of the plasma membrane and the occurrence of oxidative stress (Mou et al., 2019), resulting in rupture of the outer mitochondrial membrane, reduction or disappearance of the mitochondrial cristae, and condensation of the mitochondrial membrane, resulting in cell death (Xie et al., 2016). Recently, ferroptosis has also been proven to be involved in cancer immunotherapy. Due to its nonapoptotic nature, ferroptosis-based cancer therapy is expected to remedy the shortcomings of traditional therapies mediated by the apoptotic pathway (Liang et al., 2019). Therefore, screening ferroptosis-related genes (FRGs) based on clinical samples is beneficial for the diagnosis of glioma and provides possible therapeutic targets.

Long noncoding RNA (lncRNA) refers to a type of noncoding RNA more than 200 nucleotides in length. lncRNAs are involved in a wide range of cellular mechanisms, from almost all aspects of gene expression to protein translation and stability (Schmitz et al., 2016). Subsequent studies found that lncRNAs are dysregulated in tumors (Srikantan et al., 2000; Ji et al., 2003; Diederichs 2014). With the continuous in-depth understanding of lncRNAs, researchers have successively discovered the effect of lncRNAs on cancer, for example, lncRNAs can change epigenetics in glioma (Pop et al., 2018). Recent studies have found that lncRNAs can control the occurrence and development of tumors by affecting the process of ferroptosis. LINC00336 as a competing endogenous RNA inhibits ferroptosis in lung cancer (Wang et al., 2019; Mao et al., 2019), and the lncRNA GABPB1-AS1 regulates erastin-induced ferroptosis through GABPB1 in HepG2 hepatocellular carcinoma (Qi et al., 2019). Furthermore, there are 3 different ferroptosis-related lncRNAs(FRL) signatures were observed to be associated with glioma prognosis, containing 15 ferroptosis-related lncRNAs, 14 ferroptosis-related lncRNAs and 9 ferroptosis-related lncRNAs, respectively (He et al., 2021; Zheng et al., 2021; Shi et al., 2022). In machine learning models, a consistent cutoff value for different datasets enhances generalizability, and thereby increasing applicability in real world. Unfortunately, the previous studies didn’t explore the best cutoff value for different datasets, which might lead to potential false positive results. Moreover, gene mutation has been proved to be an important factor affecting the survival and prognosis of glioma patients (Suzuki et al., 2015; Arita et al., 2020). For example, IDH mutation status has been shown to be closely related to the prognosis of glioma patients (Pirozzi and Yan 2021). The previous studies have not explored this aspect, too.

In this study, we obtained RNA-seq data from TCGA and GTEx databases and finally obtained three differentially expressed FRLs (DEFRLS) for constructing prognostic models. Then, the reliability of the model was verified by survival analysis, receiver operating characteristic (ROC) curve analysis and independent prognostic analysis. In addition, the mechanism of action of FRLs in glioma was further explored by gene set enrichment analysis (GSEA), mutated gene analysis, immune infiltration analysis and chemotherapeutic drug sensitivity analysis. Finally, our results provide a good predictive model and possible therapeutic targets for glioma patients.

## 2 Materials and methods

### 2.1 Data acquisition

A total of 1,850 samples from gliomas (GBM, LGG) in The Cancer Genome Atlas (TCGA) website (https://portal.gdc.cancer.gov/) and Genotype-Tissue Expression (GTEx) website (https://www.gtexportal.org) were collected, including 698 tumors and 1,152 normal samples. Then, the data were log_2_-processed, and Ensembl IDs were converted to official gene symbols. lncRNAs and protein-coding genes were screened by the Genome Reference Consortium Human Build 38 (GRCh38).

### 2.2 Identification of ferroptosis-related lncRNAs

The ferroptosis-related dataset (FerrDb) was obtained from FerrDb (http://www.zhounan.org/ferrdb/index.html) website, resulting in a total of 176 validated human FRGs. Subsequently, Spearman correlation analysis (|*R* ^2^| > 0.6 and *p* value < 0.001) was performed according to the expression profiles of FRGs and lncRNAs, and 433 FRLs were obtained.

### 2.3 Differential expression analysis

The limma package (Ritchie et al., 2015) was used to perform differential analysis on the lncRNA expression matrix of LGG/GBM and normal samples, and a total of 2056 differentially expressed lncRNAs (DELs) were obtained. The criteria for DElncRNAs were |log_2_ (fold change) | >1 and a false discovery rate (FDR) < 0.05 (Tu et al., 2020).

### 2.4 Construction of ferroptosis-related prognostic signature

A total of 433 FRLs intersected with 2056 DElncRNAs, and 52 lncRNAs were ultimately obtained. Then, univariate Cox analysis was performed based on the “survival” R package to define potential prognostic FRLs (*p* < 0.001), and a total of 35 prognosis-related lncRNAs were obtained. A total of 611 patients were randomly divided into training or validation groups in a 1:1 ratio. Subsequently, these prognostic candidates were included in least absolute shrinkage and selection operator (LASSO)-Cox regression analysis. Finally, by choosing the optimal penalty parameter *λ* associated with a minimum 10-fold cross-validation to construct the prognostic FRLS, we established a three-gene optimal prognostic model. The ferroptosis-related prognostic risk score for each patient was formulated as follows:

$$Risk\;score=\sum_{1}^{n}coef_{i}*x_{i}$$

where *x*_*i*_ and *coef*_*i*_ represent the expression of each lncRNA and its corresponding coefficient, respectively. Based on the median risk score, we divided the training cohort patients into high-risk and low-risk groups. Kaplan-Meier curves were generated using the “survminer” R package with the log-rank test to compare OS between the high- and low-risk groups. ROC curve analysis was used to evaluate the prediction accuracy of FRLS by the R package “timeROC.” To assess the model feasibility, all validations were performed simultaneously in the training and validation cohorts.

### 2.5 Functional enrichment analysis

Differentially expressed genes (DEGs) (|log_2_ (fold change)| > 1 and FDR<0.05) between the high-risk and low-risk groups were identified using the “edgeR” (Robinson et al., 2010) R package and functionally annotated based on Gene Ontology (GO) and with the “clusterProfiler” (Wu et al., 2021).

R package of Kyoto Encyclopedia of Genes and Genomes (KEGG) (adjusted *p* value < 0.05).

### 2.6 Gene set enrichment analysis

To explore molecular and biological differences in high/low risk groups, the KEGG and HALLMARK gene sets in the Molecular Signature Database (https://www.gsea-msigdb.org/GSEA/Msigdb) were obtained. Gene set enrichment analysis (GSEA) between the two groups was performed by the “ClusterProfiler” R package (*p* < 0.05 and FDR < 0.25) (Subramanian et al., 2005). Subsequently, single-sample GSEA (ssGSEA) was performed on several representative genomes by the “GSVA” R package.

### 2.7 Assessment of immune cell infiltration and immune microenvironment

Immune infiltration in glioma patients was assessed using the ESTIMATE algorithm by the R package “estimate.” The 22 immune cell subsets obtained from the CIBERSORT portal (http://CIBERSORT.stanford.edu/) were defined using CIBERSORT’s LM22, and the differences in the infiltration of 22 immune cells were subsequently assessed using the CIBERSORT algorithm. Finally, Pearson correlation analysis was used to calculate the differences in the expression levels of immune cell markers between the two groups of patients (Tan et al., 2020).

#### 2.7.1 Cell lines

Normal human astrocytes (HA 1800) and GBM cell lines U251, LN229, KNS-89, and T98G were purchased from .Cell lines were cultured in standard culture conditions (37°C, 95% humidity, 5% CO_2_) in the culture in DMEM (Gibco BRL, United States) medium containing 10% fetal bovine serum (Gibco BRL, United States).

#### 2.7.2 Construct stable cell lines

The shRNA targeting LINC01426 (sh#1, sh#2) from Cao et al. (2020).The sh#1, sh#2 and negative control (NC) viruses were obtained from Tsingke Biotech (Tsingke, China). Subsequently, U251 and KNS-89 were infected and selected after 48 h with 2 μg/ml puromycin (cat# A1113803, Thermo Fisher).

#### 2.7.3 RNA extraction and quantitative real-time polymerase chain reaction

Total RNAs were extracted from cells by (cat# AG21024, Accurate Biology, China) following the manufacturer’s instructions. 1,000 ng of total RNA was reverse transcribed with (cat# 11139ES10, Yeasen). Gene expression was quantified by Roche LightCycler 480 using SYBR Green Master Mix (cat#Q711-02,Vazyme). GAPDH was regarded as the reference gene. All primers are from Tsingke Biotech (Tsingke, China), and the primer sequences are shown in **List 1**.

#### 2.7.4 Cell counting kit-8 assay

The cells were detached with 0.25% trypsin, centrifuged, and resuspended in complete culture medium at a density of 5 × 10^4^ cells/ml. Each well of the 96-well plates was administered 100 μl cell suspension. Subsequently, at 24, 48, 72, and 96 h, the CCK8 kit was used for detection. After cultivating for 0, 24, 48, and 72 h, 10 μl CCK-8 solution (cat#A311-01, Vazyme) was added. Finally, the proliferation rate of the cells was detected by absorbance at 450 nm. All of the CCK-8 assays were repeated three times with the similar results and data represented with mean ± SD.

#### 2.7.5 Reactive oxygen species detection

The levels of intracellular ROS were detected using a reactive oxygen species detection kit (cat# 50101ES01, Yeasen) following the manufacturer’s instructions.Flow cytometer recording fluorescence intensity.

#### 2.7.6 Determination of malondialdehyde and Fe^2+^ levels

MDA test kits (cell samples, E-BC-K028-M, Elabscience), are used to determine levels of MDA. Detection of Fe^2+^ levels by FerroOrange probe (F374, Dojindo).

### 2.8 Statistical analysis

R software (version 4.1.0) was used for all statistical analyses and graphical visualizations. Spearman correlation analysis was used to analyze the correlation between FRGs and FRLs. The proportion of tumor-infiltrating immune cells between the high- and low-risk groups was analyzed by the Wlicox test. The chi-square test was used to analyze the differences in clinical characteristics such as age and sex between the two groups. Cox univariate regression analysis and multivariate Cox regression analysis were used to define independent prognostic factors for OS in the two groups. Time-dependent ROC curve analysis was used to assess the predictive accuracy of the OS prognostic models. Two-tailed *p* < 0.05 was considered statistically significant.

## 3 Results

### 3.1 Identification of ferroptosis-related differentially expressed LncRNAs in glioma patients

**FIGURE 1:**
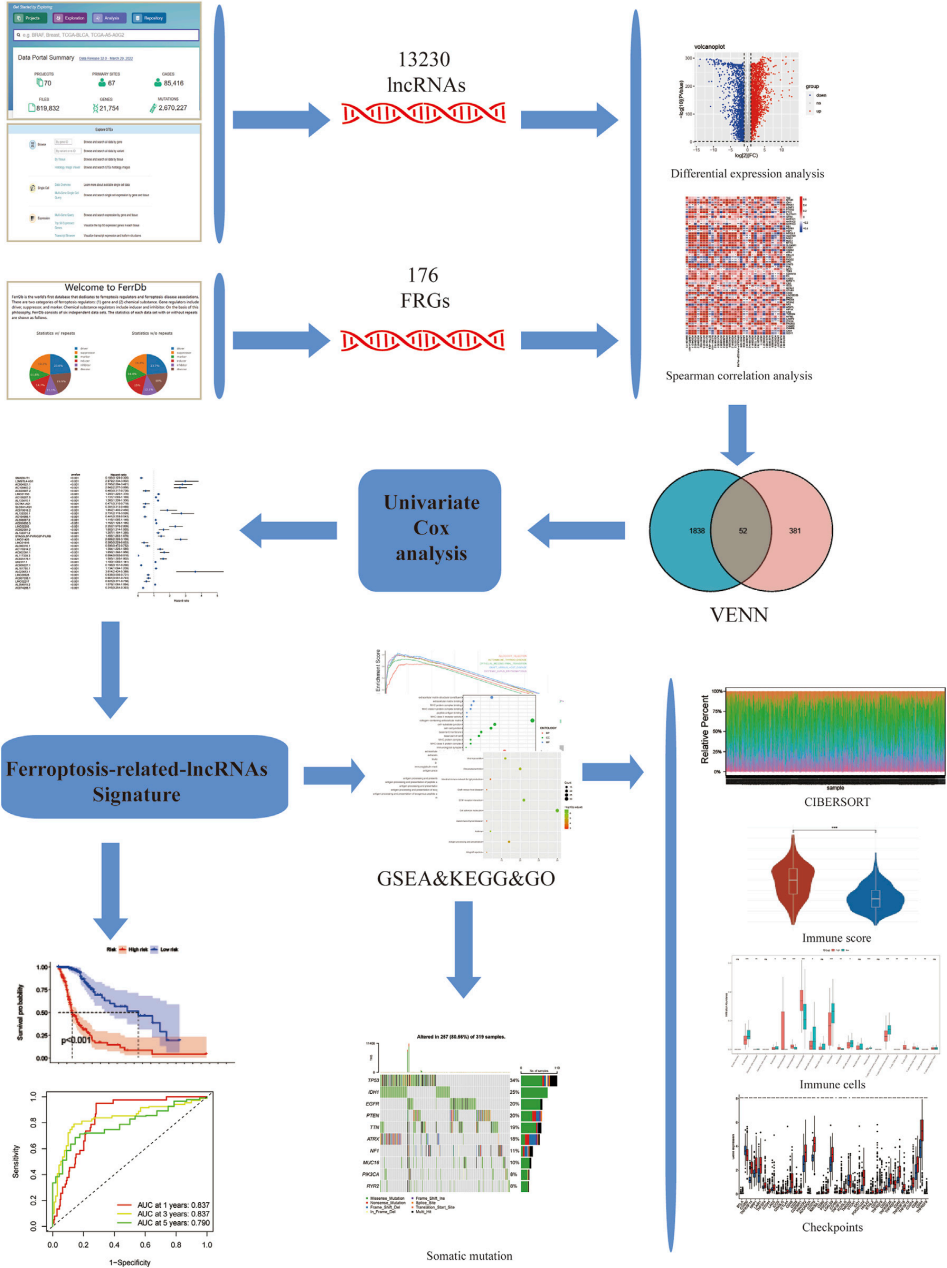
Study flowchart. 13,230 LncRNAs were obtained from TCGA and GTEx databases. 176 ferroptosis-related genes (FRGs) were obtained from the FerrDb database. Then, 433 ferroptosis-related lncRNAs (FRLs) were identified according to Spearman correlation analysis. Next, univariate COX analysis was applied to construct a 3-FRL signature. Finally, GSEA, KEGG, GO analysis, immune correlation analysis, somatic mutation analysis were applied to determine the potential function of this feature.

**FIGURE 2:**
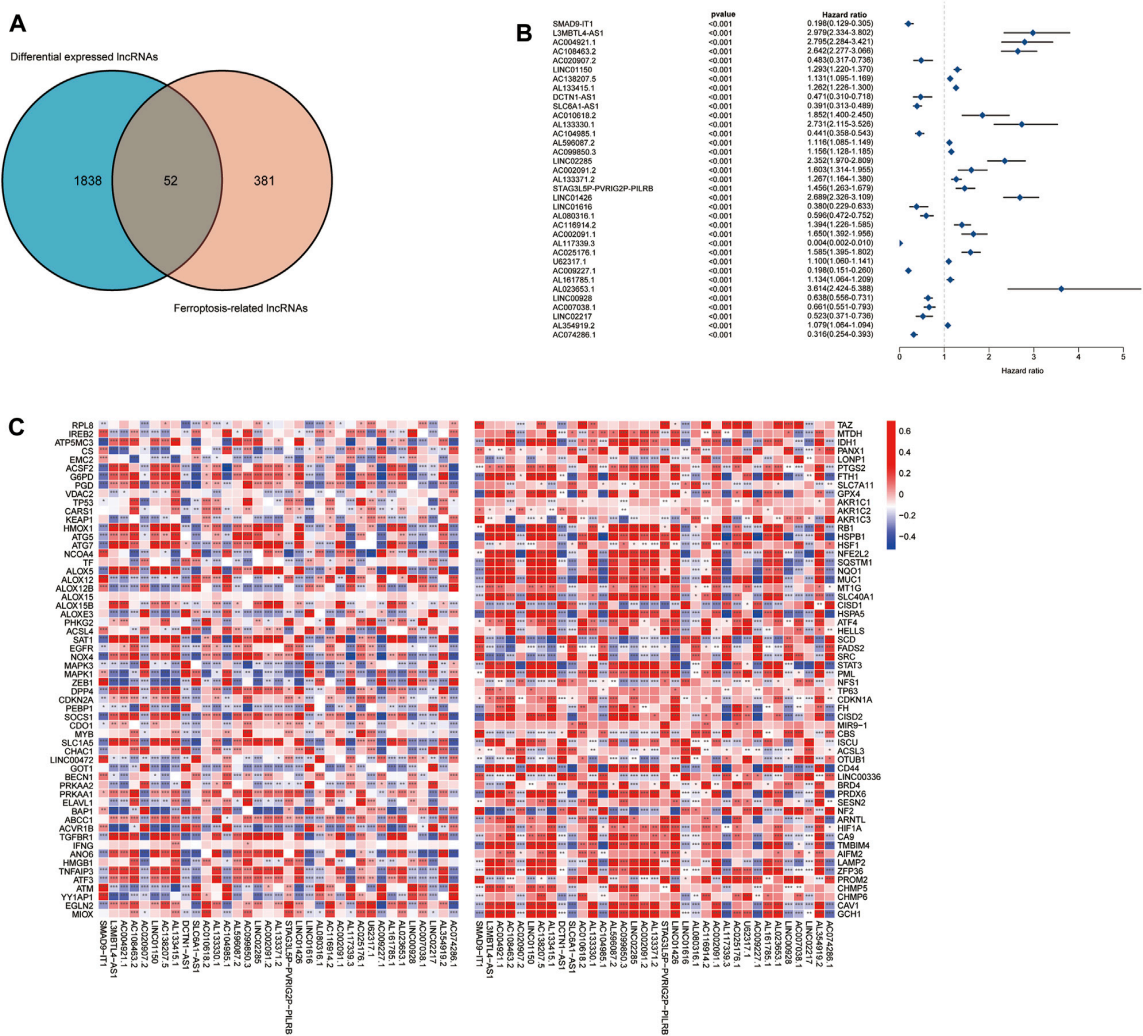
Prognostic analysis of differentially expressed ferroptosis-related lncRNAs. **(A)** Differentially expressed FRLs obtained from differentially expressed lncRNAs and FRLs by Venn diagram. **(B)** Forest plots showing the results of the Cox univariate regression analysis approximately 35 prognostic differentially expressed FRLs. **(C)** The correlation between 35 prognostic FRLs and 176 FRGs in the TCGA-LGG/GBM cohort. The colour of each unit shows the degree of corelation. *p < 0.05, **p < 0.01, and ***p < 0.001.

The complete flow chart of the study is shown in Figure 1. We collected a total of 1,850 samples, of which 698 tumor samples (GBM, LGG) were from the TCGA database (https://portal.gdc.cancer.gov/repository), and 1,152 normal samples were from the TCGA and GTEx databases (https://www.gtexportal.org/home/datasets). A total of 13,230 lncRNAs were identified. Furthermore, based on the known ferroptosis-related dataset Ferrdb (http://www.zhounan.org/ferrdb/), we obtained 176 ferroptosis-related genes (FRGs). The specific details of these genes are recorded in Supplementary Table S1. To obtain ferroptosis-related lncRNAs (FRL), Spearman correlation analysis was conducted between lncRNAs in the TCGA database. An FRL was identified if it was significantly correlated with one or more FRGs (|*R* ^2^| > 0.6 and *p* < 0.001). In total, 433 FRLs were defined. We used a PCA map and bar plots to show the distribution of those samples, as shown in Supplementary Figures S1A,B. Then, we compared the expression of lncRNAs in tumor and normal tissues from the TCGA-GTEx database (log_2_| FC| > 1, FDR < 0.05) and identified 1,890 DELs, including 1,132 upregulated DELs and 758 downregulated DELs. We used volcano plots to show these data in Supplementary Figure S1C. Finally, we identified 52 ferroptosis-related DELs (FRDELs) between FRLs and DELs (Figure 2A).

### 3.2 Identification of prognostic ferroptosis-related differentially expressed lncRNAs

To further understand the prognostic potential of FRDELs, after obtaining OS data for GBM and LGG in TCGA, we predicted the prognostic potential of 52 FRDELs using univariate Cox regression analysis. Finally, 35 prognostic ferroptosis-related differentially expressed lncRNAs (PFRDELs) were obtained (*p* < 0.001) (Figure 2B; Supplementary Figure S1D). The coexpression relationship between the 35 PFRDELs and 176 FRGs is shown in Figure 2C. Thirteen PFRDELs were considered protective factors, and 22 were considered risk factors (the list of these lncRNAs is shown in Supplementary Table S2).

### 3.3 Construction and validation of a ferroptosis-related lncRNAs prognostic model

To check the prognostic value of these DEFRLS. We collected clinical data from TCGA-GBM/LGG and randomly divided them into two groups: a training group and a validation group. The clinical characteristics of those samples in the above two groups are shown in Table 1.

**TABLE 1:** The clinical characteristics of glioma patients in the training and validation group.

Clinical parameters	Group		*p* Value
	Training	Validation	
Gender			
Female	265	260	>0.05
Male	182	188	
Age (years)			
≤65	362	364	>0.05
>65	85	84	
Grade			
Stage II	120	123	>0.05
Stage III	132	131	
Stage IV	195	194	
IDH Status			
Mutant	219	217	>0.05
Wild type	228	231	

**FIGURE 3:**
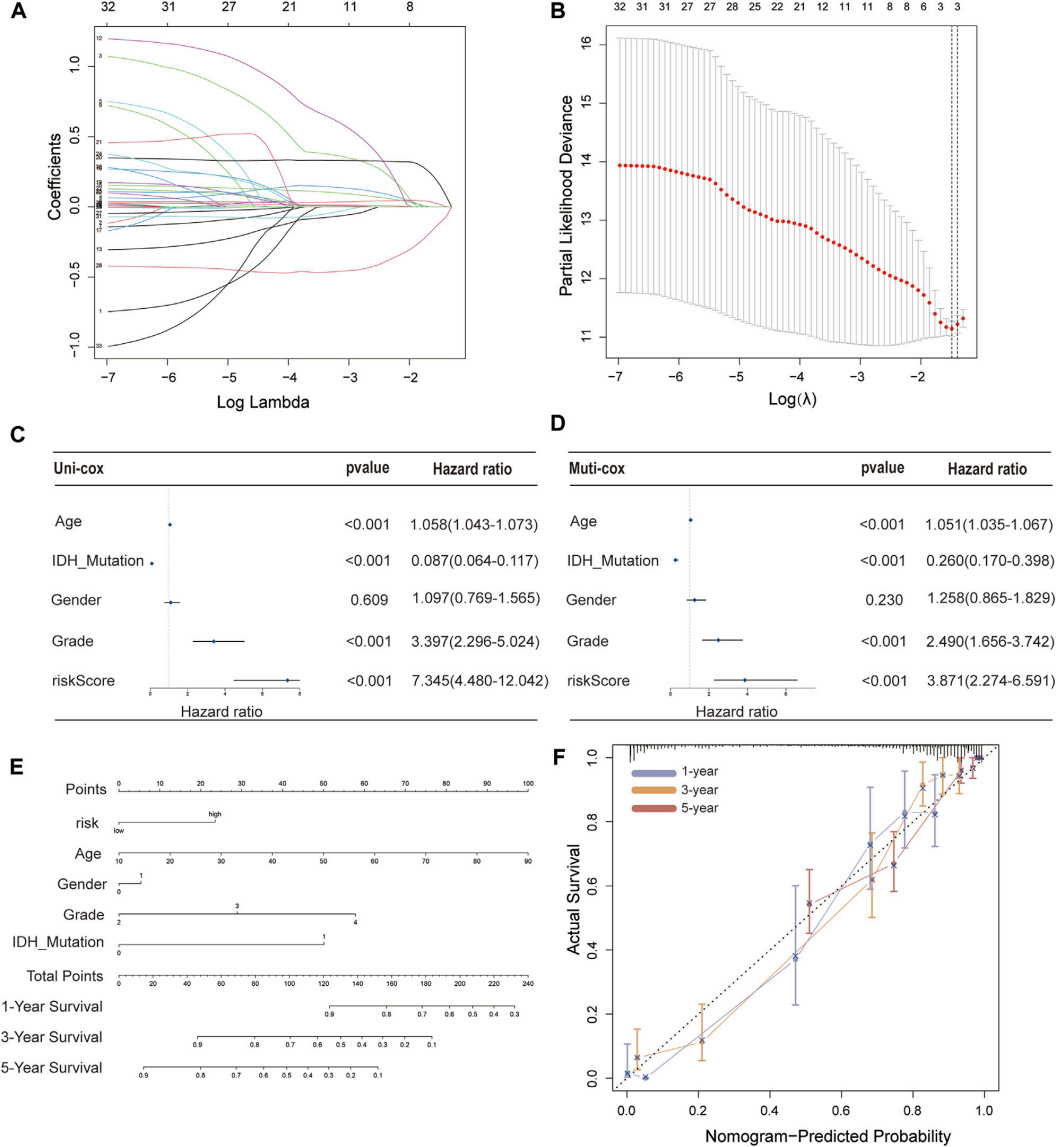
Construction of a 3-FRL signature and the analysis of independent prognostic potential. **(A,B)** The least absolute shrinkage and selection operator (LASSO) regression was performed with the minimum criteria. **(C)** Results of the univariate Cox regression analysis and multivariate Cox regression analysis regarding OS of the 3-FRLs signature. **(D)** Nomogram of OS over time for glioma patients. **(E)** This calibration curve is used to assess the accuracy of the nomogram model, and the dashed line represents the ideal nomogram. **(F)** The calibration curve for evaluating the accuracy of the nomogram model. The dashed diagonal line in grey colour represents the ideal nomogram.

These 35 PFRDELs in the training group were incorporated into the least absolute shrinkage and selection operator (LASSO) regression. As a result, 3 PFRDELs stood out for the construction of the prognostic FRLS, including AL133415.1, LINC01426 and AC009227.1. Then, based on the optimal penalty parameters (*λ*) of the LASSO model, a prognostic risk evaluation model for 3-FRLs was constructed. The cvfit and lambda curves are shown in Figures 3A,B. In this model, each patient with GBM/LGG in the TCGA database was calculated with a risk score by summing the product of the expression level of each selected ferroptosis-related lncRNA and the corresponding coefficient. [Risk Score = AL133415.1*0.01732 + LINC01426*0.15269 + AC009227*(−0.10944)].

**FIGURE 4:**
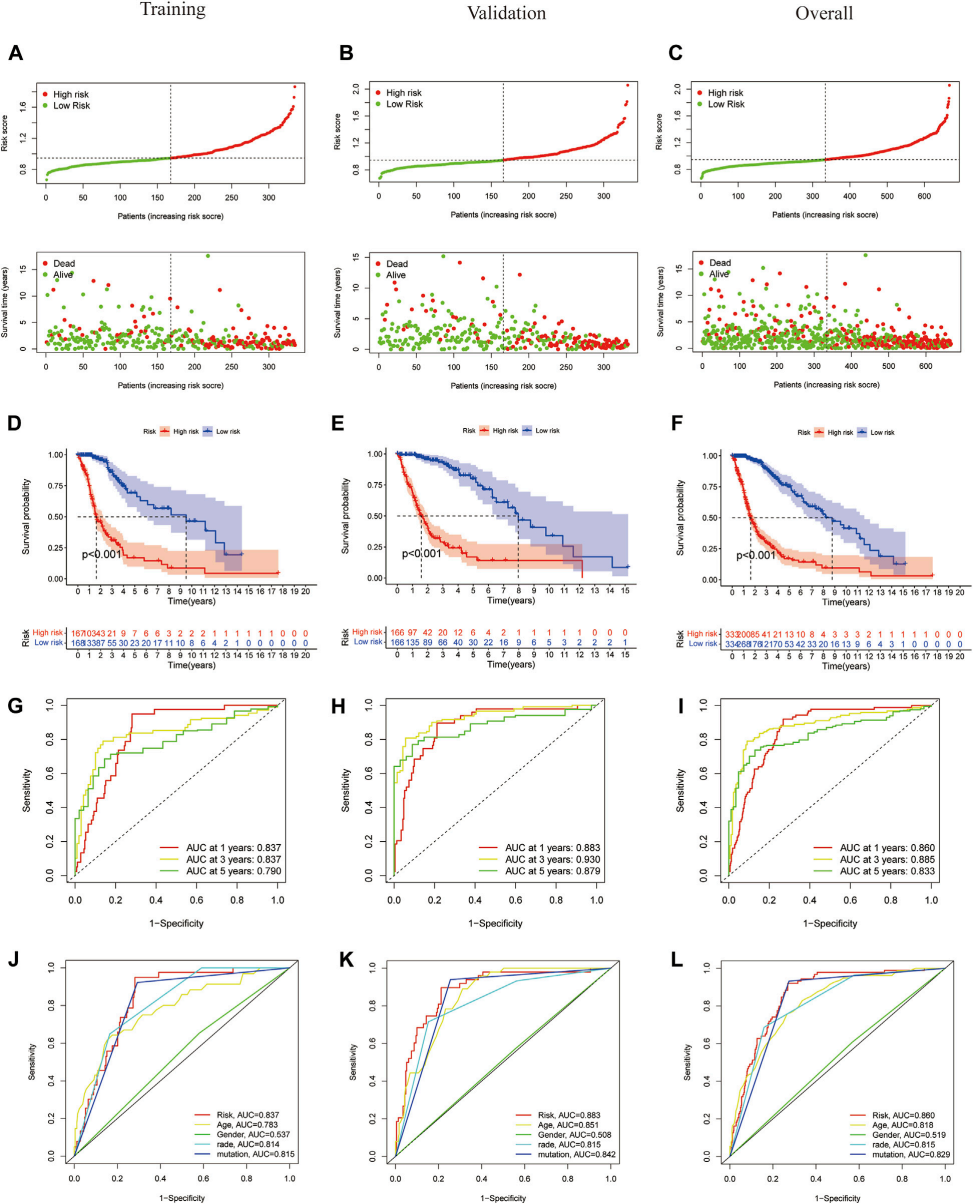
Construction and validation of 3-FRLs models in training cohorts, validation and overall groups. **(A–C)** The distribution plots of the risk score and survival status in training cohorts, validation and overall groups. **(D–F)** The Kaplan-Meier curves for survival status and survival time in the training, validation and overall groups. **(G–I)** The receiver operating characteristic (ROC) curve analyses of the prognostic FRLS in predicting 1-, 3-, and 5-year overall survival in training cohorts, validation and overall groups. **(J–L)** Risk scores and other prognostic features of the 3-FRLS model were compared using AUC of ROC curves in the training cohort, validation and overall groups.

To evaluate the independent predictive potential of this signature, the OS-related factors were identified by univariate and multivariate Cox regression analyses. The results of both univariate and multivariate Cox regression analyses indicated that the PFRDLS-based risk score was always an independent prognostic factor for the OS rate of GBM/LGG patients (Figures 3C,D). Predictive nomograms were then constructed and the associated factors’ scores on the scales were summed to calculate the likelihood of survival for these patients. The 1-, 3- and 5-year OS rates could be predicted accurately when compared with those of the ideal predictive model (Figures 3E,F). To evaluate the prognostic value of this 3-FRLs model. Then, the samples in the training group were stratified into the high-risk group and low-risk groups using the median risk score as the cutoff value. Subsequently, the risk score distribution and OS status distribution of the above two samples were determined, and the results showed that the distributions of the two samples were reasonable (Figure 4A). Kaplan-Meier analysis of the samples showed that the OS rate of GBM/LGG patients in the high-risk group was worse than that in the low-risk group (Figure 4D). Then, an ROC curve was performed in the training group and found that the prognostic accuracy of the 3-FRLs model was better than that of other clinicopathological characteristics. Since both disease state and factor values change over time, a time-dependent ROC curve was also performed in the training group. The AUCs for 1-, 3-, and 5-year OS in the training group were 0.837, 0.837, and 0.790, respectively (Figure 4G). We then constructed ROC curves for comparison with other clinicopathological features (Figure 4J). To determine whether the prognostic significance of FRLS persisted in other groups, the validation group and overall group were validated in heatmaps, distribution figures, Kaplan-Meier survival analysis and time-dependent ROC analysis. The distribution of the above two risk group samples in the validation group and the overall group is shown in Figures 4B–L. Since molecular subtype and IDH state contribute to the outcome and classification of glioma patients, it is hard to exclude the bias of these factors. Therefore, we performed the Kaplan-Meier analysis to verify whether the signature is functional in both LGG and GBM/IDH wt and IDH mut patients. The results show that the signature is functional in patients with LGG and IDHmut. However, GBM and IDH wt patients did not have statistical significance because of the large difference in sample size (Supplementary Figures S2A–D).All results agree that the mortality rate of the low-risk groups is lower than that of the high-risk groups, and the FRLS prognosis can accurately and stably predict the survival outcome of GBM/LGG patients.

### 3.4 Relationship between the 3-FRLs signature and the clinicopathological characteristics in glioma patients

**FIGURE 5:**
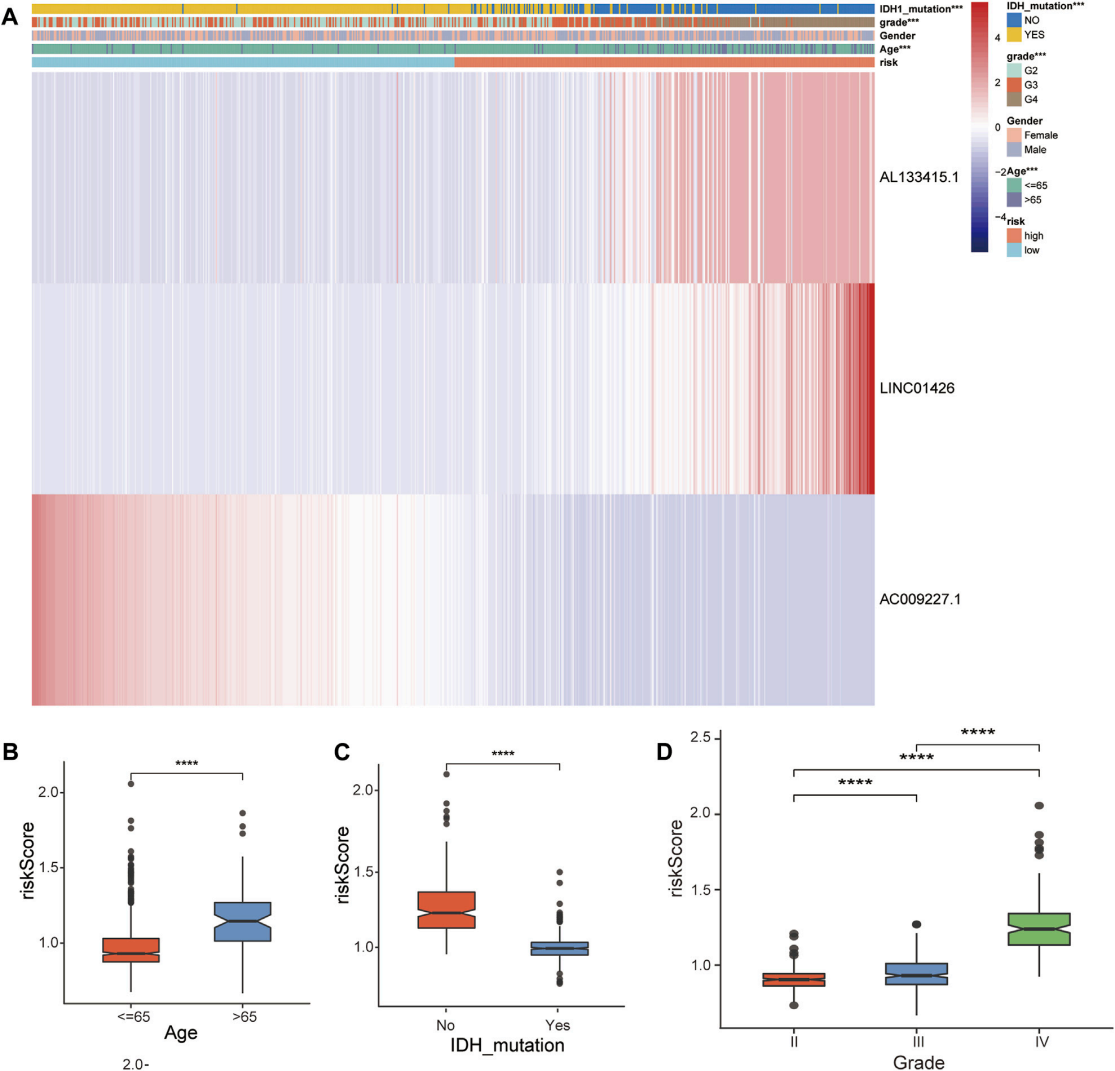
Correlation analysis between the prognostic FRLS and clinicopathological characteristics in the TCGA cohort. **(A)** Heatmaps depict the distribution of FRLS expression levels and clinicopathological features in high-risk and low-risk groups. **(B–D)** Different risk score levels in glioma patients stratified by age, sex, grade, IDH mutation status. *p < 0.05, **p < 0.01, ***p < 0.001, and ns No significance.

In the TCGA-GBM/LGG cohort, two lncRNAs in our model were considered risk lncRNAs and upregulated in the high-risk group. Only AC009227.1 was considered a protective lncRNA that was upregulated in the low-risk group (Figure 5A). Next, we compared the differences in clinical characteristics between the two risk subgroups in terms of age, sex, glioma grade, and IDH status. We found that with the increase in glioma grade, the expression of risk lncRNAs was upregulated, and the expression of protective lncRNAs was downregulated, which ultimately led to the improvement of the risk score. Similar results were observed for age and IDH status, and the clinical features are also compared in Figures 5B–D. Studies have shown that the status of IDH has a significant relationship with the prognosis of glioma (Yan et al., 2009). Taken together, these results suggest that our 3-lncRNA signature has a significant potential to predict the prognosis of glioma patients by assessing risk scores through correlated gene expression levels.

### 3.5 Discovery of molecule function and pathways by GESA, gene ontology and kyoto encyclopedia of genes and genomes analysis

**FIGURE 6:**
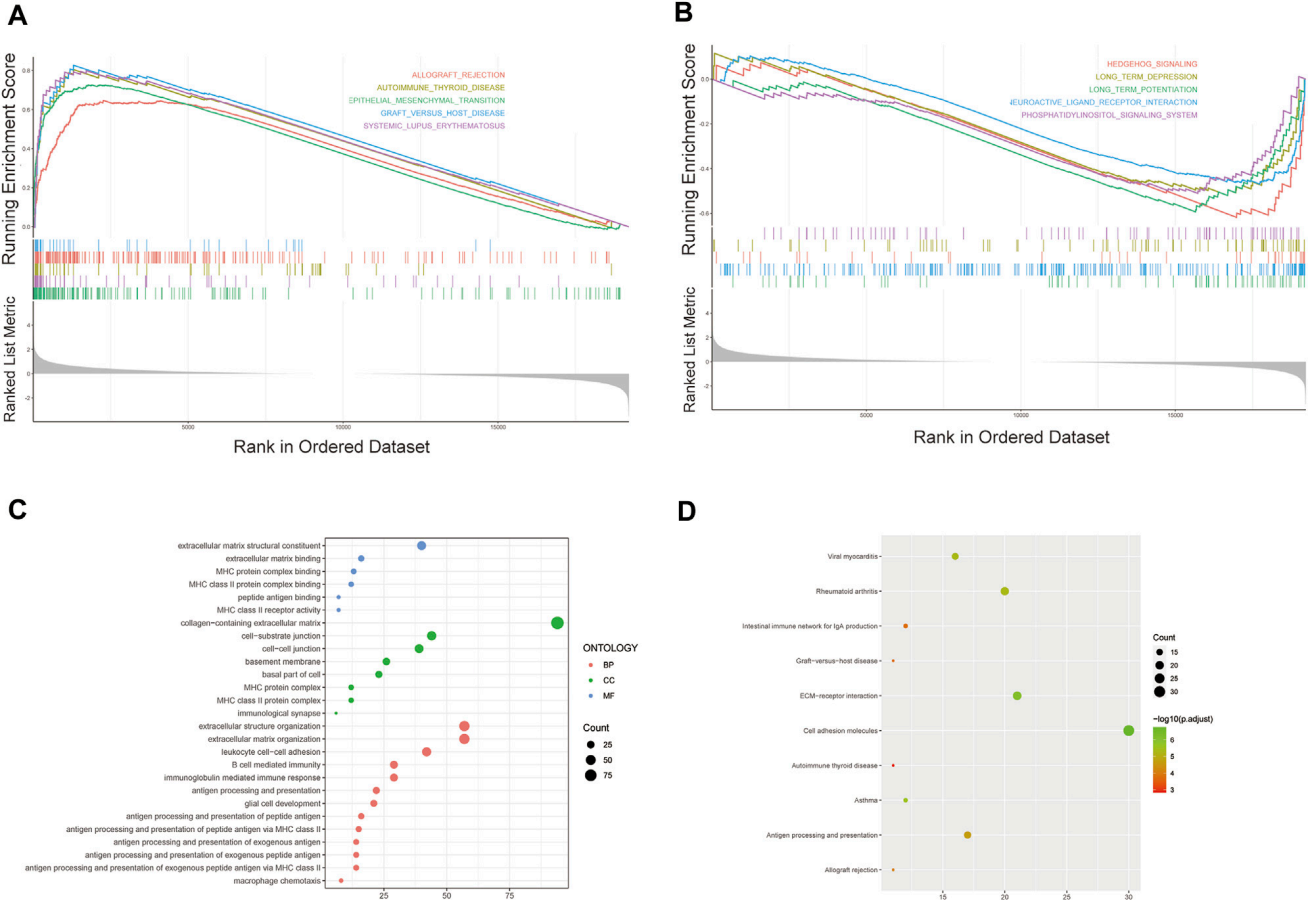
Gene biological function and pathway enrichment analysis of prognostic signatures of FRLs in high-risk group and low-risk group. **(A)** GSEA shows significant enrichment of immune-related and metastasis-related pathways in high-risk glioma patients. **(B)** GSEA shows significant enrichment of cancer-related signaling pathways in low-risk glioma patients. **(C)**GO analysis revealed enrichment of many immune-related processes and tumor metastasis-related processes. **(D)**KEGG analysis revealed that many immune-related processes and tumor metastasis-related processes were enriched.

We further performed GSEA to explore potential differences in biological functions and signaling pathways between different risk groups classified by the 3-FRL signature. Many tumor metastasis pathways are enriched in high-risk populations, such as epithelial-mesenchymal transition (EMT). At the same time, many immune-related pathways were also enriched in high-risk groups, such as systemic lupus erythematosus, autoimmune thyroid disease, graft versus host disease and allograft rejection (Figure 6A). In addition, many signaling-related pathways were enriched in the low-risk group, such as the calcium signaling pathway, phosphatidylinositol signaling system, and hedgehog signaling (Figure 6B). Interestingly, some pathways related to metabolism and proliferation, such as angiogenesis-related pathways, glutathione metabolism and drug metabolism (amino sugar and nucleotide sugar metabolism), were also enriched. The details of the GSEA results are listed in Supplementary Table S3. To explore the biological functions characterizing DEGs between different risk groups. DEGs between the high-risk group and the low-risk group were determined by the cutoff of log_2_|FC| > 1 and FDR < 0.05, and annotation GO enrichment analysis and KEGG pathway analysis were then performed (*p* < 0.05). GO analysis shows enrichment of biological process (BP), molecular function (MF), and cell component (CC) in Figure 6C. Expectedly, the GO analysis revealed a significant enrichment of immune-related functions, especially in relation to MHC protein complex binding, immunological synapse and antigen processing and presentation of peptide antigen. Similarly, the KEGG analysis indicated the enrichment of metastasis-related pathways, including cell adhesion molecules and ECM−receptor interactions. In addition, many immune-related pathways were significantly enriched, including antigen processing and presentation, rheumatoid arthritis and asthma (Figure 6D). The above two bioinformatics analyses are similar to the GSEA results. In conclusion, these results suggested that the risk score of the 3-FRLs signature was associated mainly with tumor immunity, tumor metastasis and biological metabolism in glioma.

### 3.6 Immune-related analysis of glioma patients using the prognostic signature

**FIGURE 7:**
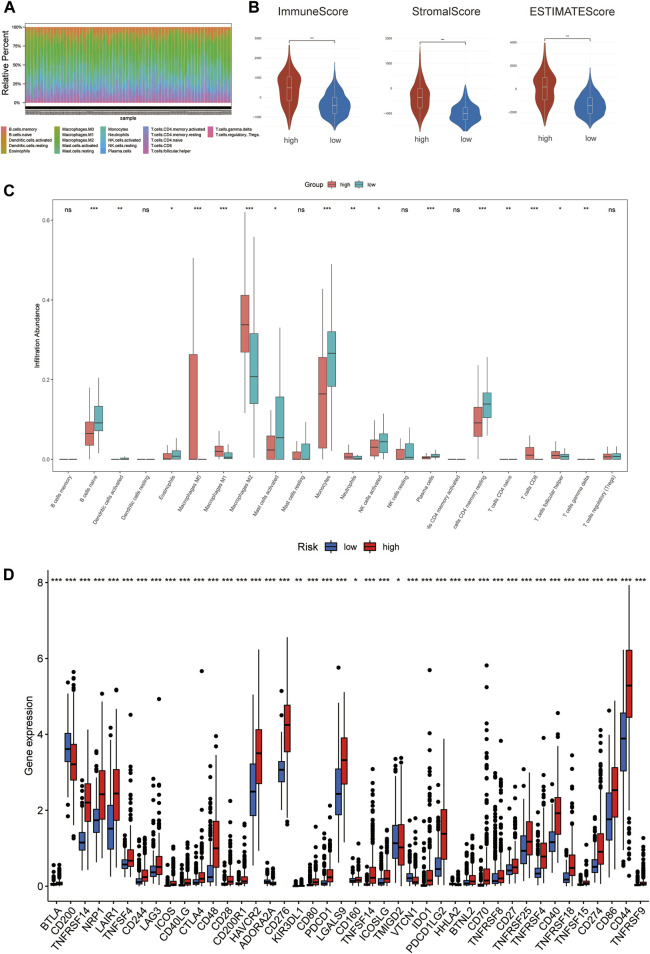
The degree of immune infiltration in glioma patients. **(A)** Immune cell distribution in high-risk and low-risk groups in the 3-FRLs model. **(B)** Stroma, immune, and ESTIMATE scores in the high-risk and lowrisk groups in glioma patients. **(C)** Boxplot of comparison of immune cells in high-risk and low-risk groups. **(D)** Boxplots comparing immune checkpoint genes in high- risk and low-risk groups.

To investigate the correlation of ferroptosis-related features and antitumor immunity in glioma patients. We used the CIBERSORT algorithm to identify the immune cell infiltration landscape of all patients with GBM/LGG from the TCGA database and calculated the proportion of each typical immune cell (Figure 7A). We compared the differences in immune cells in the low-risk group and high-risk group from the stromal score (substrate cells in the tumor tissue), immune score (immune cell infiltration in the tumor tissue) and estimate score (the summation of stromal and immune scores from individual cases and defined as tumor purity). The results showed that the scores of the high-risk group were higher than those of the low-risk group (*p* < 0.001) (Figure 7B). Meanwhile, there were differences in the proportion of each immune cell between the high-risk group and the low-risk group, including memory B cells, naive B cells, resting dendritic cells, eosinophils, activated mast cells, monocytes, neutrophils, activated NK cells, resting NK cells, plasma cells, CD4 memory activated T-cells, naïve CD4 T-cells, CD8 T-cells, gamma delta T-cells, regulatory T-cells and M0, M1, and M2 macrophages (Figure 7C). Meanwhile, we found statistically significant differences in 42 checkpoint genes between the high- and low-risk groups (Figure 7D). Among these genes, 39 genes, including PDCD1 (PD-1), CD274 (PD-L1), CTLA4 and LAG3, were highly expressed in the high-risk group, many of which are validated effective immunotherapy targets. In addition, only CD200 had lower expression than the low-risk group (Pardoll 2012; Anderson et al., 2016; Postow et al., 2018; Li et al., 2019). In conclusion, by comparing the relationship between risk scores calculated from 3-FRLs signatures and immune infiltrating cells, the results suggest that the risk level of glioma patients is related to immune cell infiltration.

### 3.7 Cancer-related gene mutation in the 3-FRLs signature

**FIGURE 8:**
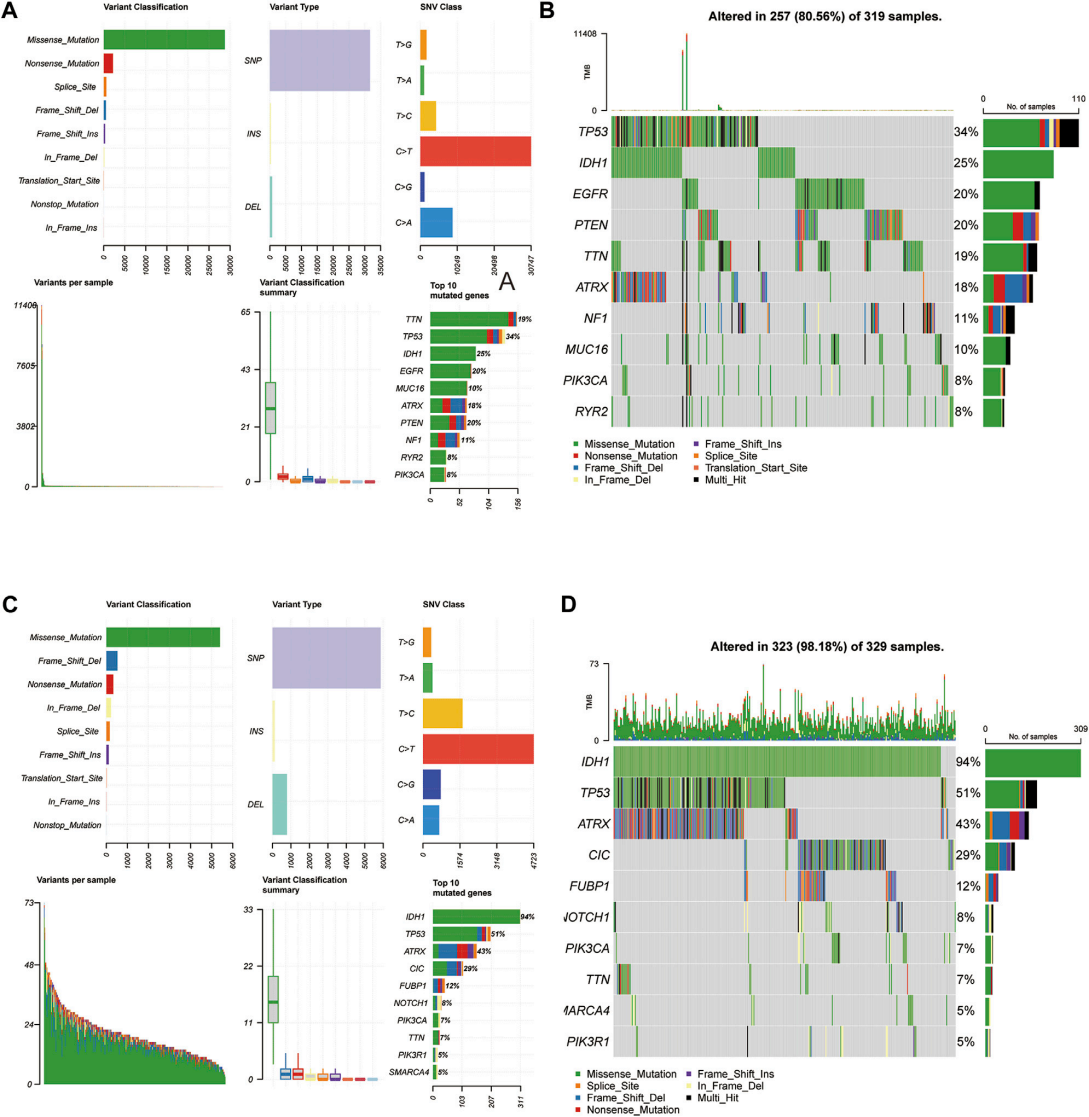
Somatic mutation analysis in high-risk and low-risk groups. **(A,B)** MAF-summary plots and oncoplots of somatic mutations in high-risk groups. **(C,D)** MAF-summary plots and oncoplots of somatic mutations in low-risk groups.

Mutations arise from replication errors or from DNA damage that is either repaired incorrectly or left unrepaired. The transition from normal cells to tumor cells is often accompanied by genetic mutations. The rates of different mutational processes vary among tumors and cancer types (Martincorena and Campbell 2015).Therefore, to further analyze whether the gene mutation levels of the 3-FRLs signature differed, we sorted out cancer-related gene mutations between the high-risk and low-risk groups separately (Figures 8A–D). Genes such as TP53 (34%), IDH-1 (25%), EGFR (20%), PTEN (20%), TTN (19%), and ATRX (18%) had the top six mutation frequencies in the high-risk group. IDH-1 (94%), TP53 (51%), ATRX (43%), CIC (29%), FUBP1 (12%), and NOTCH1 (8%) were the top six genes with the highest mutation frequencies in the low-risk group. In conclusion, IDH-1 (25% vs. 94%), TP53 (34% vs. 51%), and ATRX (18% vs. 43%) had relatively lower mutation rates in the high-risk group. However, mutations in these genes have been shown to be more frequently found in patients with low-grade gliomas, further demonstrating the predictive power of the 3-FRLs signature for glioma patients.

#### 3.7.1 Validation of ferroptosis-related lncRNAs expression and LINC01426 regulated erastin-induced ferroptosis

**FIGURE 9:**
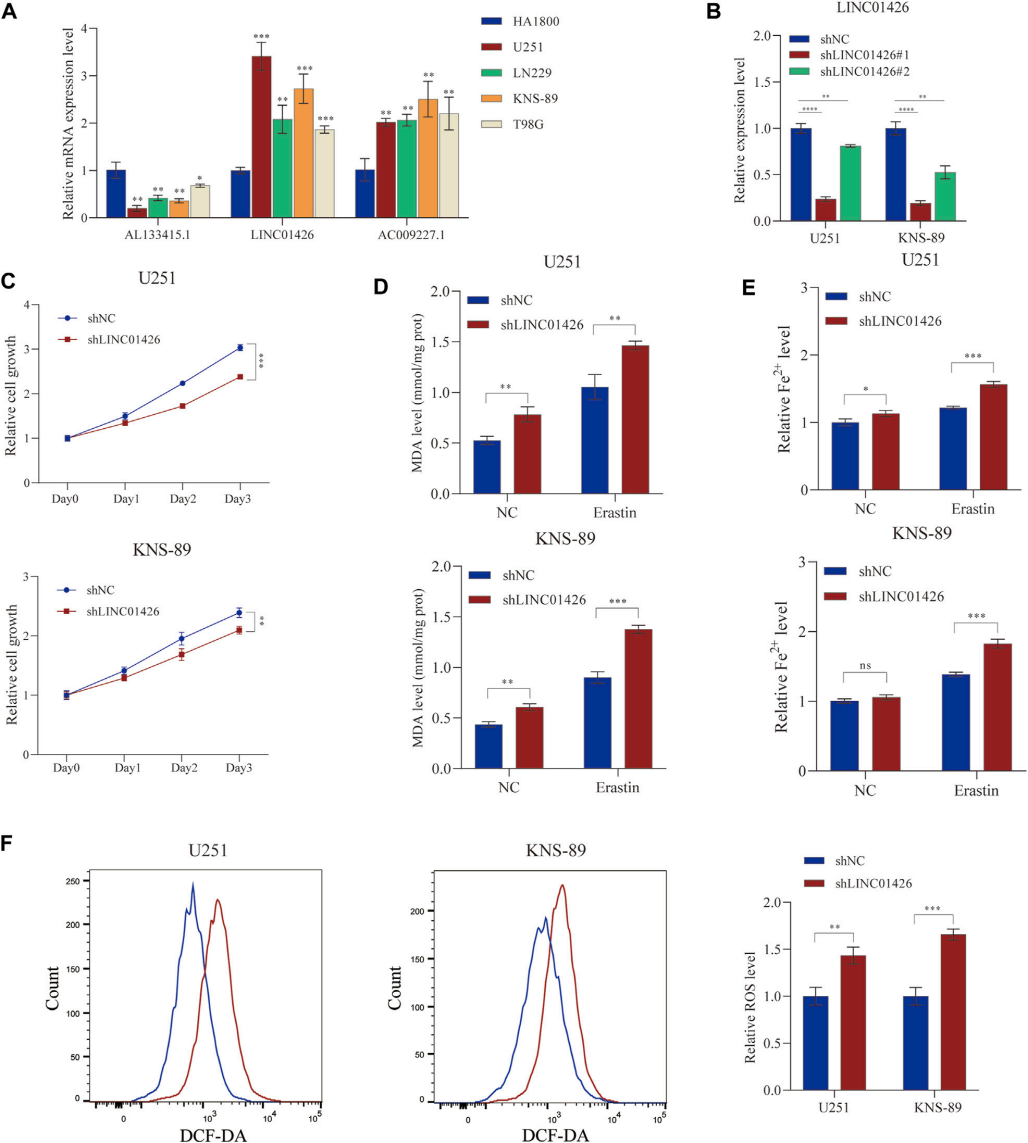
Validation of the expression level of the 3-FRLs in cell lines and ferroptosis regulation. **(A)** Expression analysis of 3-FRLs in four glioma cell lines (U251, LN229, KNS-89, T98G) with HA1800 lines (normal astrocytes). **(B)** Relative expression level of LINC01426 after transfection with the corresponding shRNA. **(C)** Cell proliferation levels of U251 and KNS-89 after knocking down LINC01426. **(D,E)** The ferroptosis process was evaluated by detecting MDA and Fe^2+^ levels in the non-erastin-induced and erastin-induced groups. **(F)** The comparison of erastin-induced ROS in the treatment and control groups.*p < 0.05, **p < 0.01, ***p < 0.001, and ns, No significance.

PhyloCSF is a comparative genomics method to distinguish protein coding and non-coding regions (Lin et al., 2011). Therefore, we used PhyloCSF to determine whether these FRLs have protein-coding ability. As shown in Supplementary Figure S2E, LINC01426 with negative scores was retained as potential noncoding RNAs (Wang et al., 2020), AL133415.1 and AC009227.1 may have the potential to encode short peptides. Thus, these FRLs do not have the ability to encode complete proteins. We further observed the expression levels of these FRLs in cell lines, as shown in Figure 9A, compared with HA 1800, AL133415.1, and LINC01426 were expressed at relatively higher levels in glioma cell lines (including U251, LNS229, KNS- 89, and T98G), but AC009227.1 exhibited the opposite trend. These results further verified the correctness of the above bioinformatics research (Supplementary Figure S1D). Subsequently, we chose the LINC01426 with the highest scoring coefficient to further analyze. We use the short hairpin RNAs to achieve the stable knockdown of LINC01426 in U251 and KNS-89 . The qRT-PCR results of knockdown efficiency are shown in Figure 9B. As shown in Figure 9C, the Cell Counting Kit-8 (CCK8) assay indicates that the knockdown of LINC01426 significantly inhibited cell proliferation in U251 and KNS-89 cells.In order to further study the impact of LINC01426 on Ferroptosis, we use MDA and FerroOrange assay kits to detect malondialdehyde (MDA) and Fe^2+^ level. As shown in Figures 9D,E, after processing of 12 μM erastin (ferroptosis activator), compared with the control group, the knockdown of LINC01426 have a significant increase in the MDA and Fe^2+^ levels in U251 and KNS89 cells. The occurrence of ferroptosis has a close relationship with the accumulation of ROS (Li et al., 2020). ROS levels were clearly observed after U251 and KNS-89 cells were treated with 12 μM erastin. The erastin-induced ROS level has a significant increase after the knockdown of LINC01426 (Figure 9F). In conclusion, all results suggest that LINC01426 can inhibit the occurrence of ferroptosis in glioma.

## 4 Discussion

The reason for the high malignancy and drug resistance observed in glioma has been found to be that these tumors can effectively evade ferroptosis. Currently, many studies on glioma have focused on the relationship between lncRNAs and ferroptosis (Deng, et al., 2020; Huang et al., 2021a). The identification of FRLs is essential for finding potential therapeutic targets. However, the exploration of FRLs in gliomas is still limited. Therefore, it is important to construct a predictive model of ferroptosis-related lncRNAs. In this study, we analyzed glioma tumor samples and normal samples from TCGA and GTEx databases and obtained DELs. Then, the 176 ferroptosis-related genes obtained from the online FerrDb database were intersected, and the differentially expressed FRLs were finally screened. Subsequently, we obtained the clinical information and FRL expression profile of each patient from the TCGA database. The results identified 35 prognostic FRLs. Finally, we established a risk assessment model based on 3 FRLs. Compared to ROC curves of published literature (He et al.2021; Shi et al. 2022), our signature has more predictive ability of prognosis and contains fewer lncRNAs. The lncRNA prognosis assessment kits that have been commercialized at present are composed of only 3-5 lncRNAs (Patent No. CN201710998995.9; No. CN201810764922.8). Therefore, our signature is more clinically feasible and has potential for clinical translation.

Interestingly, in our constructed 3-ferroptosis-related lncRNA signature, LINC01426 is an oncogene that has been validated by many cancer researchers. LINC01426 promotes the development of lung cancer (Dai et al., 2020; Han et al., 2020; Liu et al., 2021; Zhu et al., 2022), clear cell renal cell carcinoma (Jiang et al., 2021), and osteosarcoma (Zhang et al., 2021). Furthermore, the role of LINC01426 in glioma has also received increasing attention, including sponging miR-345-3p and upregulating VAMP8 to promote glioblastoma (Cao et al. 2020). Mechanistic investigation showed that LINC01426 exhibited its tumor promoter role by modulating the PI3K/Akt signaling pathway (Wang et al., 2018) and PI3K has been shown to be targeted therapy for glioma (Cruceru et al., 2013). However, how LINC01426 is involved in regulating ferroptosis still needs further exploration. This study indicates that the ferroptosis process of glioma cells was inhibited after knocking down the expression of LINC01426, which fills the gap in this field.

In addition, a novel prognostic 3-lncRNA model was created. Compared with many other identified signatures, this model contains only 3 lncRNAs. Clinically, the model also has good predictive power for patient outcomes. We divided glioma patients into a high-risk group and a low-risk group based on their risk scores calculated by the formula of this prognostic model. To further explore the mechanism by which this signature regulates gliomas, we performed GSEA. The results showed that cancer metastasis pathways, such as epithelial-mesenchymal transition (EMT) and ECM-receptor interaction, were highly ranked in the high-risk group. Among these pathways, EMT can not only enhance tumor invasiveness but also be associated with enhanced stem cell properties and drug resistance (Aiello and Kang 2019). Angiogenesis-related pathways and glutathione metabolism are also enriched; angiogenesis (the formation of new blood vessels) has been shown to be an integral part of cancer development (Viallard and Larrivée 2017), and glutathione is an important component against reactive oxygen species (ROS), which are key substances in the ferroptosis process (Liu et al., 2022). Interestingly, many immune-related pathways were also enriched, including primary immunodeficiency, IL6-JAK-STAT3 signaling and the IL2-STAT5 pathway. Although the relationship between ferroptosis and the immune microenvironment remains controversial (Friedmann Angeli et al., 2019), it is reasonable to hypothesize that there is a link between ferroptosis and tumor immunity in glioma. Subsequently, KEGG enrichment analysis and GO enrichment analysis were also performed, including BP, MF and CC, and the enrichment pathway results were similar to the GSEA results. As we all know, the major barriers to effective treatment of GBM are their high proliferation, progressive spread, and invasiveness, but the underlying mechanisms for controlling gliomas are still far from understood (Groothuis 2000).Taken together, we can infer from the above results that the high-risk group suppressed the occurrence of ferroptosis through immune- and metabolic-related pathways.

Previous studies have shown that ferroptosis is closely related to tumor immunity (Xie et al. 2016; Tang et al., 2019), but direct evidence of the connection between ferroptosis and antitumor immunity was not available until Wang et al., 2019a reported that CD8^+^ T cells induce ferroptosis in tumor cells *in vivo* (Wang et al., 2019b, Green et al., 2019). In addition, studies have shown that the increased intratumor production of prostaglandin E2 (PGE2) facilitates tumor evasion of immune surveillance (Kalinski 2012; Veglia et al., 2019). In terms of immunotherapy, studies have shown that CD8^+^ T-cells are involved in radiotherapy-induced ferroptosis in human fibrosarcoma cells and melanoma cells (Lang et al., 2019). However, no study has reported a direct link between ferroptosis and immune cell infiltration in glioma. Since GSEA was enriched in many immune-related pathways, we further calculated the proportions of different types of tumor-infiltrating immune cells in gliomas. The high-risk group had higher immune, stromal, and estimated scores, as calculated by CIBERSORT from the TCGA database. Compared with the low-risk group, the high-risk group had higher expression levels of CD8^+^ T-cells and macrophages and lower expression levels of monocytes or dendritic cells. High immune and stromal scores and high macrophage infiltration are associated with poor prognosis, which is consistent with our results (Deng et al., 2020). Subsequently, we found that among the immune checkpoints, forty genes, including PDCD1 (PD-1), CD274 (PD-L1), CTLA4, and LAG3, were highly expressed in the high-risk group. Therefore, these patients might benefit from many immune checkpoint blockades (Cristescu et al., 2018), which also provides a possible modality for ferroptosis immunotherapy in the future (Tang et al., 2020).

Next, we analyzed the cancer-related gene mutation status of the two risk subgroups to further explore the relationship between risk scores and cancer-related gene mutations. We found that mutations in IDH-1, TP53 and ATRX were significantly different between the two groups. Numerous studies have shown that IDH1 mutations lead to better overall survival in glioma patients and a better response to therapies (Yan et al. 2009; Franceschi et al., 2021). Recently, an ATRX-deficient genetically engineered glioma model demonstrated that loss of ATRX reduces median survival and increases genetic instability (Koschmann et al., 2016). Moreover, TP53 mutations are frequent in low-grade gliomas and secondary glioblastomas derived therefrom (Ohgaki and Kleihues 2005). These studies have shown that mutations in certain key genes in glioma have a greater impact on prognosis. In our 3-FRLs signature, the risk scores are also strongly associated with the mutation status of these genes. Interestingly, lncRNA has recently been shown to be required for maintaining genomic stability (Lee et al., 2016; Munschauer et al., 2018).In addition, the lncRNA signatures of genome instability can Predict Survival in Patients (Huang,et al., 2021b; Xie, et al., 2016).The studies could be corroborated with our results. Not only that, our signature provides new genes for studying the relationship between lncRNAs and gene mutation. In the future, we can use these genes to explore the effect of the PUMILIO protein or the topoisomerase complex, as researchers did with NORAD.

## Data Availability

The datasets presented in this study can be found in online repositories. The names of the repository/repositories and accession number(s) can be found in the article/Supplementary Material.

## Supplementary material

SUPPLEMENTARY FIGURE S1 Identifification of differentially expressed ferroptosis related lncRNAs in GBM/LGG. **(A,B)** The PCA map and the bar plots showing the distribution of glioma samples. **(C)** The volcano map of lncRNAs differentially expressed in GBM/LGG. **(D)** The heatmap of these 35 lncRNAs showing the expression level of each lncRNA in each patient.

SUPPLEMENTARY TABLE S1 The details of all of the ferroptosis-related genes.

SUPPLEMENTARY TABLE S2 The table of differentially expressed lncRNAs and ferroptosis-related lncRNAs.

SUPPLEMENTARY TABLE S3 The list of pathways enriched in GSEA.
