# Supplementary material for: Identification and validation of ferroptosis-related lncRNA signatures as a novel prognostic model for glioma
Source: Front Genet. 2022 Sep 20;13:927142. doi: 10.3389/fgene.2022.927142 (PMC9549413; doi:10.3389/fgene.2022.927142)
Supplement: Supplementary file 2 [file Table3.DOC]

| **Clinical Parameters** | **Group** | | **p Value** |
| --- | --- | --- | --- |
|  | **Training** | **Validation** |  |
| **Gender** |  |  |  |
| Female | 265 | 260 | >0.05 |
| Male | 182 | 188 |  |
| **Age (years)** |  |  |  |
| ≤65  ＞65 | 362 | 364 | >0.05 |
|  | 85 | 84 |  |
| **Grade** |  |  |  |
| Stage II | 120 | 123 | >0.05 |
| Stage III | 132 | 131 |  |
| Stage IV | 195 | 194 |  |
| **IDH Status** |  |  |  |
| Mutant | 219 | 217 | >0.05 |
| Wild type | 228 | 231 |  |

**Table1:** The clinical characteristics of glioma patients in the training and validation group
